# NMR shift prediction from small data quantities

**DOI:** 10.1186/s13321-023-00785-x

**Published:** 2023-11-27

**Authors:** Herman Rull, Markus Fischer, Stefan Kuhn

**Affiliations:** 1 Department of Computer Science, Tartu University, Narva mnt 18, Tartu, 51009 Tartumaa Estonia; 2 Institute for Medical Physics and Biophysics, Leipzig University, Härtelstr. 16-18, 04107 Leipzig, Sachsen Germany

**Keywords:** NMR, Chemical shift, Machine learning, Prediction, Dataset size

## Abstract

Prediction of chemical shift in NMR using machine learning methods is typically done with the maximum amount of data available to achieve the best results. In some cases, such large amounts of data are not available, e.g. for heteronuclei. We demonstrate a novel machine learning model that is able to achieve better results than other models for relevant datasets with comparatively low amounts of data. We show this by predicting $^{19}F$ and $^{13}C$ NMR chemical shifts of small molecules in specific solvents.

**Graphical Abstract:**

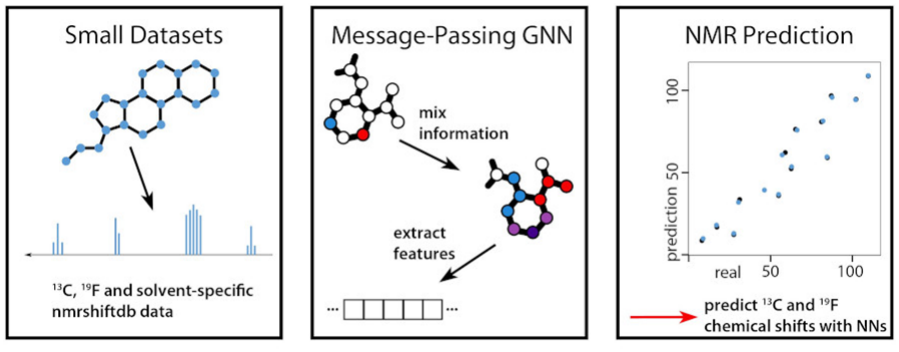

**Supplementary Information:**

The online version contains supplementary material available at 10.1186/s13321-023-00785-x.

## Introduction

Prediction of chemical shift in nuclear magnetic resonance (NMR) is a long-standing problem in chemoinformatics. [1] is perhaps the earliest publication in the field. We define prediction here as methods using existing data as opposed to ab-initio calculations. Over time, various methods for such predictions have been developed. In particular, machine learning methods have been applied, starting with early methods, like small neural networks [2], up to the latest developments in convolutional and graph neural networks. We refer the reader to the recent review [3] for an overview.

For supervised learning methods, like the mentioned neural networks, annotated datasets are needed, and the number of data points used is a significant factor in the quality of the predictions. A review of the literature shows that the datasets used are generally big, consisting of tens of thousands of molecules. Table 1 gives an overview of the number of structures used in recent publications. It should be noted, that sometimes preliminary selection was employed, e.g. the 17,000 structures of [4] are a selection (using Morgan fingerprints and the MaxMin algorithm from RDKit) from 170,000 molecules by structural diversity.

**Table 1:** Examples of papers about chemical shift prediction and the number of structures used

Literature reference	[5]	[6]	$^{13}C$ [7]	$^{1}H$ [7]	[8]	[9]	[10]	[4]
Number of structures used	32,538	57,456	21,481	10,248	75,382	400,000	8000	17,000

Unfortunately, many studies do not take the influence of the amount of training data on the quality of the prediction into account. An exception is [11], which shows that some machine learning methods only show suitable predictive power with more than 5000 training examples. However, in many practical applications, the amount of experimental data available is quite limited. Examples are NMR chemical shifts of heteronuclei, specific classes of compounds, NMR spectra measured with particular solvents, or other certain experimental conditions. For such cases of small amounts of data (defined as less than 5000 structures here), the existing models either do not provide a good solution or are yet untested on such small datasets.

In this paper, we present a graph neural network that is able to achieve good predictions with small amounts of data and is competitive with state-of-the-art models for larger amounts of data. We restrict our research to small organic molecules in solution. The prediction of solid-state NMR chemical shifts, biological macromolecules, or inorganic compounds requires different, and more specific models. Therefore, we exclude those cases from the current research.

## Materials and methods

Predictive models can be applied to many different NMR parameters, such as coupling constants, relaxation time, or peak shape. Here, we want to focus on the prediction of NMR chemical shifts. Modern (solution) NMR experiments are an excellent source of data, as they provide isotropic chemical shift information with little noise [12]. In NMR spectroscopy, the chemical shift, which is equivalent to the resonance frequency of an atom, is determined by the (chemical) environment of a nucleus. The representation of this environment is a hard task. A single atom might be represented by a vector, in which its properties are stored. However, that representation won’t work for an entire molecule. Because of this, we disregard tensor-like representation and constitute the molecule as a graph instead. There, the atoms can be described by nodes, and the connecting bonds can be described by edges. Geometric learning with graph neural networks [13, 14] provides a suitable tool to run ML algorithms on such specific structures. In the graph, information is passed along the edges, making information from connected atoms the most important information. This is equivalent to real molecules, where neighbouring atoms, which are connected with a low number of bonds, have the most impact on NMR chemical shift values.

**Fig. 1:**
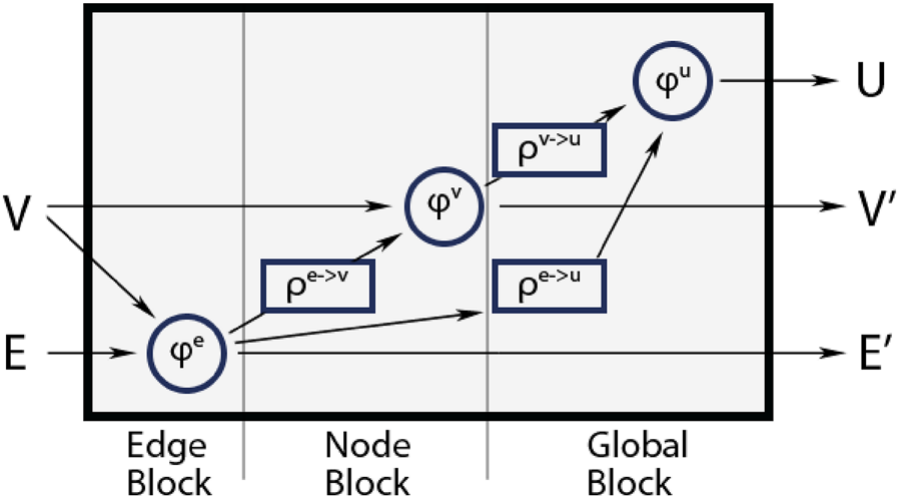
Information flow in the message-passing graph network of the 2023 model. In addition to node and edge aggregation functions, there is also an additional edge aggregation function feeding into the global update function (from [15])

For this work, we developed a model that learns the atomic properties in molecules, based on [15], which we call the “2023 model” in this paper. The model uses message-passing graph networks [16, 17], which pass information via edges in the graph, in this way building up information locally in the nodes. Following [15], we use a type of message-passing graph network block with an additional edge aggregation function, shown in Fig. 1. Since we employ the network to predict node-level features of small molecules, we disregard the second stage of the graph network described in [15]. We use a set of features which is given in Tables 2 and 3 to describe atoms and bonds.

**Fig. 2:**
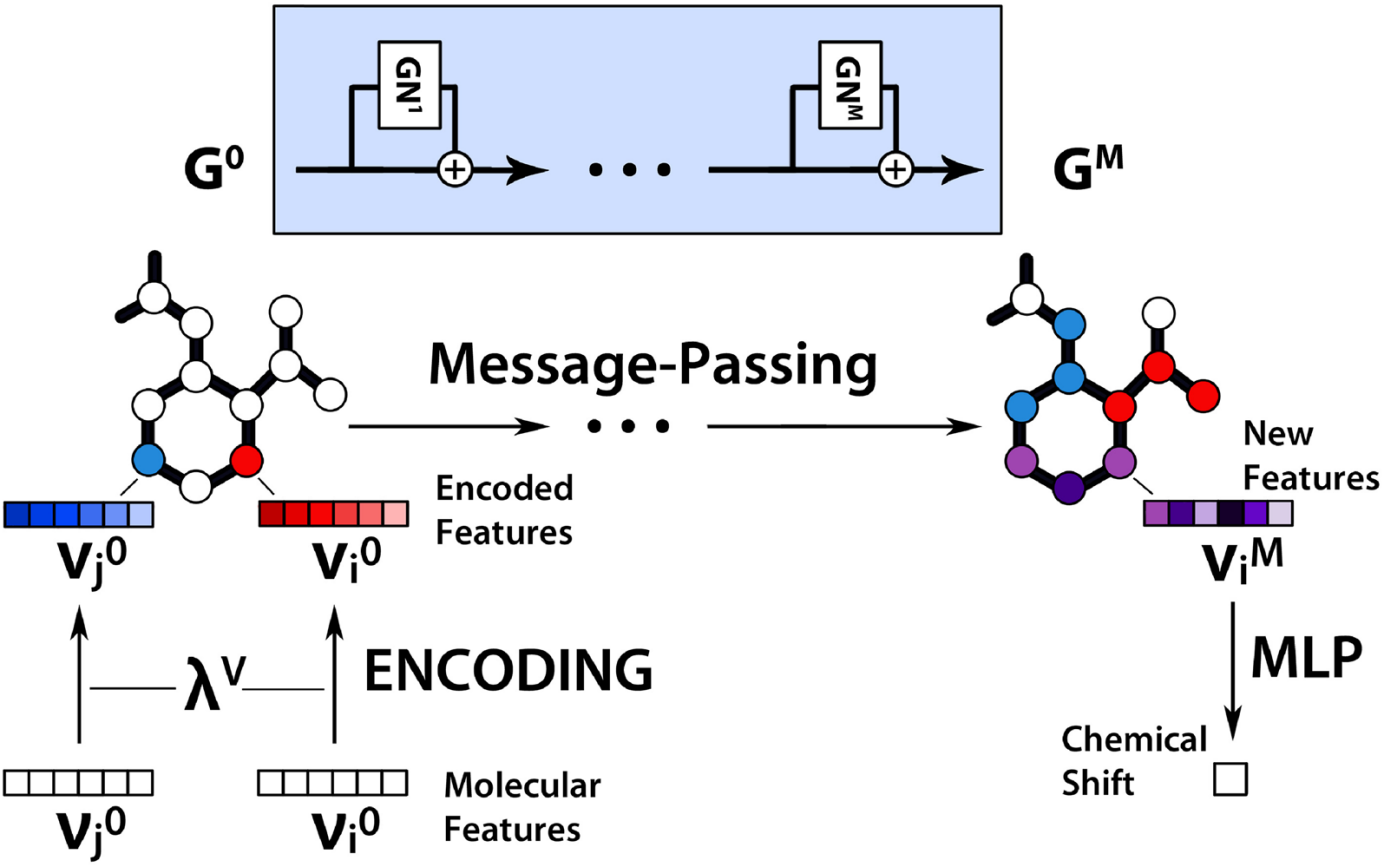
Schematic flow of information in the message-passing graph network. The workflow can be split into the following steps: encoding, message-passing, and prediction of shifts with an MLP

The flow of information is described in Fig. 2. The chosen features (see also Table 2 for edge features $\varepsilon$, and Table 3 for node features $\nu$) are encoded by the functions $\lambda ^E$ and $\lambda ^V$. These functions are represented by multi-layer perceptrons (MLPs), and encode edges ($e_i^0 = \lambda ^E (\varepsilon _i)$) and nodes ($v_i^0 = \lambda ^V (\nu _i)$) respectively. Afterward, *M* message-passing rounds are executed on the graph, using the graph network block displayed in Fig. 1. This results in new node features $v_i^M$ that get passed to another MLP that predicts the final chemical shifts.

**Table 2:** Atom features used in the 2023 model

Feature	Description [unit] (type)
Atomic number	One hot encoded [all atoms in dataset] (array[bool])
Atomic radius	Slater data from Mendeleev library [pm] (int)
Neutrons	Number of neutrons [–] (int)
Electronegativity	Pauling scale [–] (float)
Electron affinity	Value from Mendeleev library [eV] (float)

**Table 3:** Bond features used in the 2023 model

Feature	Description [unit] (type)
Bond length	Distance between atom centers [Å] (float)
Bond type	One hot encoded[single, double, triple, aromatic] (array [bool])

This specific feature selection was chosen because of preliminary experiments, which measured the impact of each feature on the final prediction. After that, the best-performing features were combined, until the prediction quality was no longer improving.

To optimize the prediction accuracy, we carefully selected the best hyperparameters for the 2023 model. The most important hyperparameters were the number of message-passing steps, the learning rate and the weight decay. The hyperparameters were optimized on the training set of $^{19} F$ data using 4-fold cross-validation. The best performing model used 6 message-passing steps, a learning rate of $10^{-3}$, and a weight decay of 0.01.

All programming is done in Python using RDKit [18] version 2022.9.5 as the main library. Furthermore, mendeleev [19] version 0.12.1 is used to calculate some atomic properties. A Jupyter notebook, containing the code and explanations, is contained in the Additional file 2 of this paper. Additional file 1 contains the same code for standalone execution.

For comparison, we use two other prediction methods. One are hierarchically ordered spherical environment (HOSE) codes [20], a long-established method that describes atoms and their environments as strings. With those, the chemical shifts of other, similar atoms are looked up and used for prediction. From the point of view of machine learning, this could be called a nearest neighbour search. The HOSE code implementation used is a port of the HOSE code implementation of the Chemistry Development Kit (CDK) [21] and available at [22]. This produces standard HOSE codes, not the stereo-enhanced HOSE codes of [23].

We use the model from [5] as a modern machine learning model, which we call the “2019 model” in this paper. This uses a convolutional graphical neural network to combine feature vectors for an atom with those of its neighbours to do the prediction. Evaluation of the methods was generally done using a 75:25 training-to-test split. We have decided against a separate validation set, due to the small size of the datasets.

All data was taken from nmrshiftdb2, an open NMR database [24]. It contains lists of chemical shift values as well as raw data of various 1D and 2D NMR experiments for a number of different nuclei. We focus on particular subsets here, as explained in the subsections of Sect. "Results". It should be noted that the datasets we used consist of random selections of structures. It might be possible to optimize the training process with small datasets by ensuring structural diversity or even distribution in chemical space. We did not follow this and assumed the random distribution of data. In particular, we include all experimental data from nmrshitdb2 (if they fit the subsets used in Sect. "Results"). This is opposed to other work, e.g. [5] where the choice is restricted to molecules with only common elements. This also explains slightly different results using the 2019 model with data from nmrshiftdb2, apart from changes to the database over time.

For comparing the performance of the models in various conditions we report three values: The mean absolute error (MAE), the root mean squared error (RMSE), the mean absolute scaled error (MASE), and the standard deviation $\sigma$ of the error. The standard deviation is calculated over all of the predictions of the model and is used to measure the amount of variation of the error from the mean. We use MASE as a scale-invariant measure, which allows comparing different nuclei and solvents.

## Results

### Overall behaviour

**Fig. 3:**
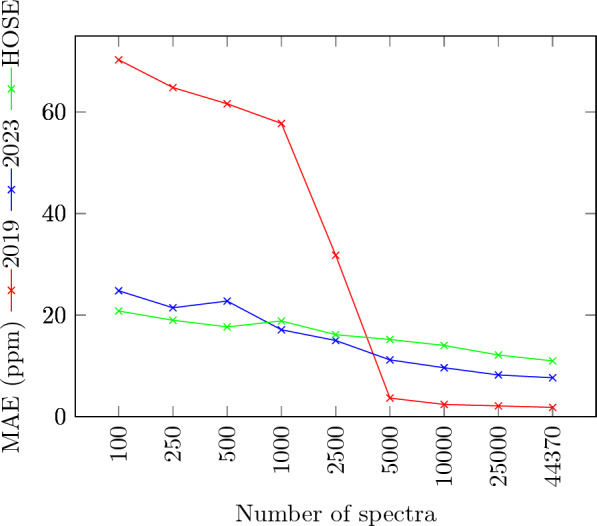
MAE of a $^{13}C$ NMR shift prediction, using increasing numbers of samples

First, we wanted to compare the new 2023 model to HOSE codes and the 2019 model. In order to do so, we analyzed the predictive performance of the different models when trained on an increasing number of molecules. The results are shown in Fig. 3 and Table 4. It can be clearly seen that the new model outperforms the 2019 model when trained on up to 2500 data points (structures), whereas the 2019 model performs better from 5000 data points onward. The sharp improvement of the 2019 model in that range was already seen in [11], and an explanation of that spontaneous improvement is yet outstanding.

The HOSE codes offer good predictive power that was previously observed in other published work, however, in some cases, a prediction based on HOSE codes is not possible, as noted in Table 4. This can happen if no examples with high enough similarity (i.e. at least one sphere) exist in the training set. The table also shows that the standard deviation of the 2019 model’s results is significantly lower than with HOSE codes, indicating that the model is more stable than the HOSE code prediction.

In order to test the influence of those molecules for which HOSE codes find no matches, we have also made predictions leaving out those molecules using HOSE codes and the 2023 model. We restricted this to those dataset sizes where both methods were very close. Table 5 shows that there is no uniform behaviour here: HOSE code predictions improve for 100, 250, 1000, 2500, and 5000 molecules, but get worse for 500 molecules. The 2023 model gives less good results for 100, 250, 1000, and 5000 structures, but improves for 500 and 2500 structures. The range where the two methods are performing similarly is unchanged.

**Table 4:** Prediction results for $^{13}C$ shifts using increasing numbers of spectra

		100	250	500	1000	2500	5000	10000	25000	44370
2019 model	MAE (ppm)	70.31	64.84	61.64	57.77	31.81	3.65	2.40	2.11	1.82
	RMSE (ppm)	83.30	79.86	76.36	70.71	36.27	5.41	3.35	4.09	3.13
	MASE	1.49	1.39	1.32	1.23	0.67	0.07	0.05	0.04	0.04
	$\sigma$ (ppm)	52.29	52.51	52.69	48.15	28.05	6.64	5.08	5.13	4.57
2023 model	MAE (ppm)	24.8	21.45	22.77	17.11	15.0	11.18	9.63	8.21	7.65
	RMSE (ppm)	46.65	40.82	44.66	45.27	40.27	32.82	29.01	25.63	24.53
	MASE	0.52	0.46	0.48	0.36	0.31	0.23	0.20	0.17	0.16
	$\sigma$ (ppm)	45.29	40.44	44.41	45.13	40.11	32.77	28.95	25.58	24.48
HOSE code	MAE (ppm)	20.81	18.99	17.68	18.85	16.14	15.2	14.02	12.14	10.98
	RMSE (ppm)	35.29	33.44	30.94	30.32	30.03	29.19	27.95	25.84	24.60
	$\sigma$ (ppm)	34.72	33.26	30.74	30.29	30.01	29.18	27.95	25.83	24.5
	MASE	0.43	0.40	0.37	0.40	0.34	0.31	0.30	0.26	0.24
	Missing predictions	17.6	19.6	26.3	33.9	41.4	51.0	57.9	76.2	85.2

**Table 5:** Prediction results for $^{13}C$ shifts using increasing numbers of spectra

		100	250	500	1000	2500	5000
2023 model	MAE (ppm)	47.58	31.50	21.33	17.76	14.13	11.99
	RMSE (ppm)	69.88	53.56	44.62	40.43	34.57	31.76
	MASE	1.01	0.64	0.43	0.36	0.28	0.24
	$\sigma$ (ppm)	65.50	51.93	44.26	39.60	34.68	31.50
HOSE code	MAE (ppm)	18.84	18.64	18.02	17.14	15.99	14.98
	RMSE (ppm)	31.49	32.43	32.02	31.01	29.92	28.94
	$\sigma$ (ppm)	30.57	32.17	31.17	30.96	29.91	28.94
	MASE	0.40	0.38	0.37	0.35	0.32	0.30

The dataset does not contain molecules where HOSE model fails to predict shifts

### Heteronuclei

$^{13}C$ and $^{1}H$ are the most popular nuclei for NMR spectroscopy, mainly due to the natural abundance of magnetically susceptible isotopes and their presence in organic compounds. Other nuclei are also used for certain applications, but the amount of data available is much smaller. Therefore, they are a good test case for our model, where we use $^{19}F$ spectra as an example. In nmrshiftdb2, there are currently 957 structures with measured $^{19}F$ spectra. We disregard the spectra that are calculated via ab-inito calculations in nmrshiftdb2 and use only one spectrum per compound in the rare case that several spectra are recorded.

We use the same machine learning models and HOSE codes as for $^{13}C$ in Sect. "Overall behaviour". It might be possible to improve the prediction by optimizing a model specifically for a nucleus, but this is not within the scope of this work. Generally, $^{19}F$ should behave similar to $^{13}C$ and $^{1}H$, which might not be the case e.g. for metals.

**Table 6:** Prediction results for $^{19}F$ shifts using increasing numbers of spectra

		100	250	500	957
2019 model	MAE (ppm)	79.43	72.68	69.65	57.82
	RMSE (ppm)	82.54	84.04	73.23	61.86
	MASE	1.21	1.51	1.64	1.36
	$\sigma$ (ppm)	47.32	93.13	43.18	41.61
2023 model	MAE (ppm)	22.25	15.94	13.32	9.77
	RMSE (ppm)	45.19	38.46	34.02	27.95
	MASE	0.34	0.33	0.31	0.22
	$\sigma$ (ppm)	43.57	37.56	33.44	27.73
HOSE code	MAE (ppm)	12.21	10.53	7.87	7.38
	RMSE (ppm)	25.97	29.68	20.16	23.33
	$\sigma$ (ppm)	25.70	29.45	20.13	23.32
	MASE	0.18	0.21	0.18	0.17
	Missing predictions	1.93	2.88	7.38	4.75

**Table 7:** Prediction results for $^{19}F$ shifts using increasing numbers of spectra

		100	250	500	955
2023 model	MAE (ppm)	16.85	11.75	10.61	7.41
	RMSE (ppm)	30.50	26.87	27.16	20.10
	MASE	0.45	0.34	0.30	0.24
	$\sigma$ (ppm)	29.54	26.34	26.96	22.65
HOSE code	MAE (ppm)	11.90	9.92	9.59	7.96
	RMSE (ppm)	25.48	24.93	25.66	25.77
	MASE	0.41	0.31	0.28	0.25
	$\sigma$ (ppm)	25.25	24.60	25.43	25.65

The dataset does not contain molecules where HOSE model fails to predict shifts

**Fig. 4:**
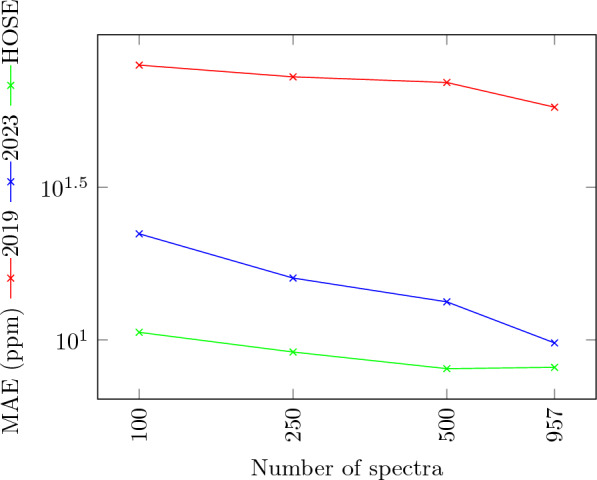
MAE of a $^{19}F$ NMR shift prediction, using increasing number of samples, on a logarithmic scale

**Fig. 5:**
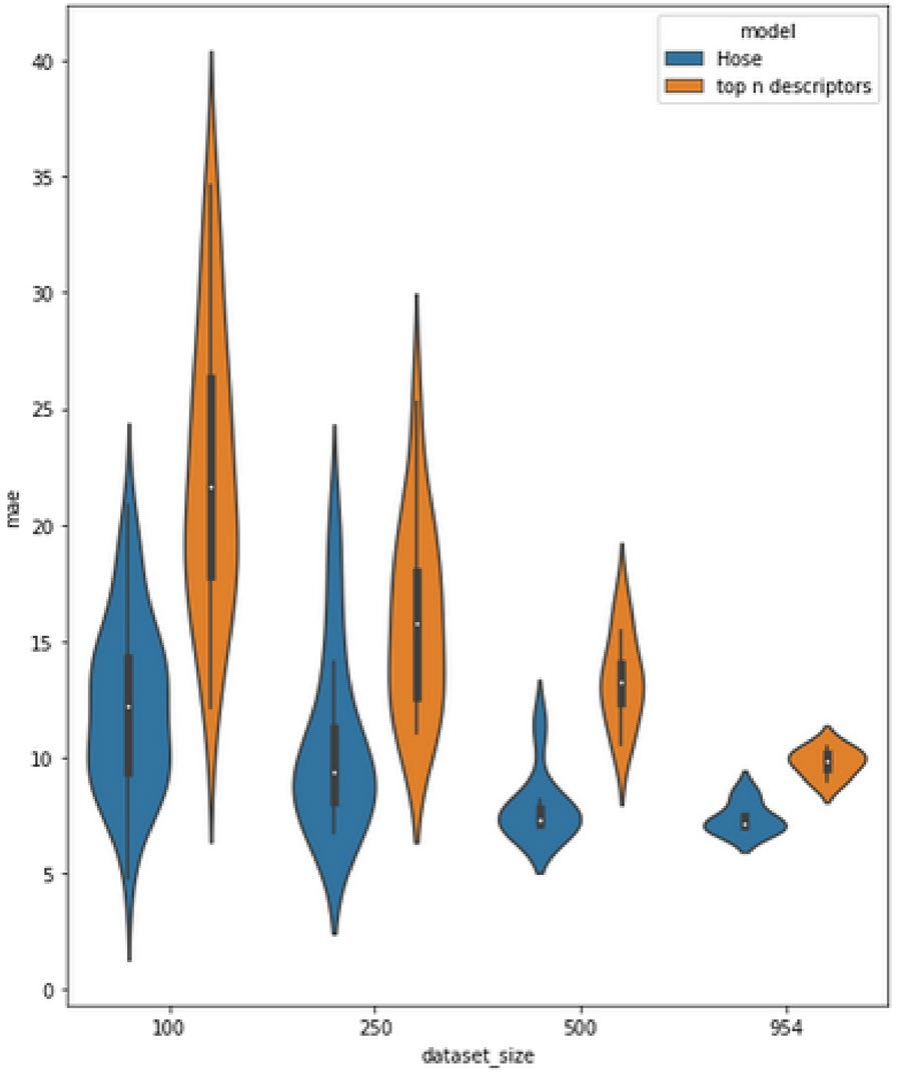
Comparison of the accuracies and their distribution of the HOSE code and GNN prediction

Table 6 and Fig. 4 show the results of the predictions based on the 957 $^{19}F$ spectra in nmrshiftdb2, using 100, 250, 500, and 957 (all) spectra for training the models. The HOSE code and 2019 model results are similar to those in [11] (differences are due to an older version of nmrshiftdb2 used in the paper), with the HOSE codes being significantly better than the 2019 model for those small amounts of data. We expect the model to improve with more data, similar to $^{13}C$, however, the amount of data is limited for this nucleus. Our new model, shown in blue in Fig. 4, outperforms the 2019 model when trained on 100 spectra and improves significantly, almost reaching the quality of the HOSE code model when trained on 957 spectra (note Fig. 4 uses a logarithmic scale). Figure 5 shows the distribution of the 2019 model and HOSE codes, showing the significant improvement with more data.

Predictions based on HOSE codes are fairly accurate but have the inherent disadvantage that they might not give a prediction at all, as discussed previously. Machine learning models will always predict chemical shifts even for less similar molecules, as they are able to generalize. Therefore, the category “Missing Predictions” is only used for the HOSE codes. We have also tested predictions leaving out molecules for which HOSE codes find no matches. The results for this are shown in Table 7. Here, with the maximum amount of data, the 2023 model gives a better result than HOSE codes do. This indicates that without those very unusual (within the dataset) molecules, the generalisation of the neural network is able to surpass the similarity search provided by HOSE codes.

### Solvents

The solvent used is one of the major factors influencing the chemical shift values of a particular compound due to its influence on the chemical environment of the molecule, the possibility of forming hydrogen bonds, changes in the charge state of the investigated molecule, and more. For prediction purposes, it is common practice to ignore the solvent (e.g. [5] or [7]). More accurate predictions would require using solvent information. One problem with this is the relatively low number of spectra for particular solvents, even for $^{13}C$ and $^{1}H$ spectra. For example, nmrshiftdb2 currently has 2324 $^{13}C$ NMR spectra in Chloroform-D1, 456 spectra in Dimethylsulphoxide-D6, and 351 spectra in Methanol-D4 (those being the most common solvents in the database).

We are using those data to train separate models for each solvent and compare the results to the values achieved by using all $^{13}C$ spectra. The results are shown in Table 8. It should be noted that the models are the same as used for the previous prediction with all solvents and the $^{19}F$ nuclei and are not optimized for a solvent-specific prediction. We can still make the following observations:

- The solvent-specific training produces much better results compared to the overall model. For example, for Chloroform-D1, the 2019 model and the 2023 model reach an MAE of 24.06 respectively 4.55 ppm with 2324 spectra, whereas with all solvents the MAE is 31.81 respectively 14.60 with 2500 spectra.
- The overall tendency is similar to what we have seen before: The predictive quality of the 2019 model starts off with high errors and significantly improves beyond 1000 spectra. The 2023 model outperforms the 2019 model on smaller datasets, due to its quick improvements when trained on up to 2500 spectra. HOSE codes are generally doing well, but do not improve much.
- The 2023 model achieves errors of less than 5 ppm with 1000 spectra for Chloroform-D1. That is better than the 2023 model with all data. For the 2019 model to become better than 5 ppm, almost 5000 spectra are needed. This means that, given the available data, our new model outperforms the 2019 model. For Dimethylsulphoxide-D6 and Methanol-D4, our new model’s results are much better than the 2019 model’s results trained on the same number of spectra.

**Table 8:** Prediction results for $^{13}C$ shifts using increasing numbers of spectra. n/a indicates that not enough data were available, numbers in brackets indicate number of compounds used, deviating from the top header

			100	250	500	1000	2324
Chloroform-D1 (CDCl3)	2019 model	MAE (ppm)	64.69	58.24	58.62	50.91	24.06
		RMSE (ppm)	80.32	74.71	74.63	66.18	34.38
		MASE	1.32	1.22	1.21	10.4	0.48
		$\sigma$ (ppm)	53.17	53.02	52.01	48.61	29.47
	2023 model	MAE (ppm)	18.89	7.47	5.23	4.32	4.12
		RMSE (ppm)	34.76	15.69	12.42	10.62	10.53
		MASE	0.38	0.15	0.10	0.08	0.08
		$\sigma$ (ppm)	31.99	15.02	12.17	10.47	10.48
	HOSE code	MAE (ppm)	5.35	4.81	4.32	4.03	3.19
		RMSE (ppm)	8.79	8.53	8.03	7.76	6.99
		MASE	0.10	0.10	0.08	0.08	0.06
		$\sigma$ (ppm)	8.77	8.51	8.02	7.76	6.99
		Missing predictions	11.92	14.23	17.30	19.42	30.25
Dimethylsulphoxide-D6 (DMSO-D6, C2D6SO)	2019 model	MAE (ppm)	91.67	93.09	85.84 (456)	n/a	n/a
		RMSE (ppm)	100.41	100.92	93.76 (456)	n/a	n/a
		MASE	2.12	2.13	1.92 (456)	n/a	n/a
		$\sigma$ (ppm)	46.28	44.75	45.01 (456)	n/a	n/a
	2023 model	MAE (ppm)	24.03	5.82	5.75(456)	n/a	n/a
		RMSE (ppm)	37.35	10.50	8.85 (456)	n/a	n/a
		MASE	0.55	0.13	0.12 (456)	n/a	n/a
		$\sigma$ (ppm)	31.26	9.92	7.61 (456)	n/a	n/a
	HOSE code	MAE (ppm)	4.96	4.27	3.65 (456)	n/a	n/a
		RMSE (ppm)	7.61	6.88	6.21 (456)	n/a	n/a
		$\sigma$ (ppm)	7.60	6.87	6.21 (456)	n/a	n/a
		MASE	0.11	0.09	0.08 (456)	n/a	n/a
		Missing predictions	9.95	12.0	10.0 (456)	n/a	n/a
Methanol-D4 (CD3OD)	2019 model	MAE (ppm)	78.60	71.92	69.41 (351)	n/a	n/a
		RMSE (ppm)	89.66	84.17	82.03 (351)	n/a	n/a
		MASE	1.84	1.66	1.60 (351)	n/a	n/a
		$\sigma$ (ppm)	49.35	49.95	49.75 (351)	n/a	n/a
	2023 model	MAE (ppm)	18.65	7.10	5.53 (351)	n/a	n/a
		RMSE (ppm)	32.69	15.31	9.94 (351)	n/a	n/a
		MASE	0.43	0.16	0.12 (351)	n/a	n/a
		$\sigma$ (ppm)	29.10	14.53	9.11 (351)	n/a	n/a
	HOSE code	MAE (ppm)	4.67	4.24	3.64 (351)	n/a	n/a
		RMSE (ppm)	8.15	8.05	7.37 (351)	n/a	n/a
		$\sigma$ (ppm)	8.13	8.04	7.37 (351)	n/a	n/a
		MASE	0.10	0.09	0.08 (351)	n/a	n/a
		Missing predictions	8.42	6.75	12.5 (351)	n/a	n/a

**Table 9:** Prediction results for $^{13}C$ shifts using increasing numbers of spectra

			100	250	500	1000	2321
Chloroform-D1 (CDCl3)	2023 model	MAE (ppm)	9.56	7.49	5.90	4.95	3.04
		RMSE (ppm)	19.79	14.90	9.60	7.59	4.27
		MASE	0.20	0.15	0.12	0.10	0.06
		$\sigma$ (ppm)	19.46	10.56	8.08	5.67	4.21
	HOSE code	MAE (ppm)	4.99	4.54	4.13	3.86	3.09
		RMSE (ppm)	7.43	7.01	6.59	6.30	5.43
		MASE	0.10	0.09	0.08	0.08	0.06
		$\sigma$ (ppm)	7.42	7.00	6.59	6.30	5.43
Dimethylsulphoxide-D6 (DMSO-D6, C2D6SO)	2023 model	MAE (ppm)	8.09	4.80	3.17(454)	n/a	n/a
		RMSE (ppm)	17.32	7.33	5.04 (454)	n/a	n/a
		MASE	0.18	0.10	0.07 (454)	n/a	n/a
		$\sigma$ (ppm)	16.62	7.03	4.98 (454)	n/a	n/a
	HOSE code	MAE (ppm)	4.74	4.08	3.61 (454)	n/a	n/a
		RMSE (ppm)	7.18	6.42	5.99 (454)	n/a	n/a
		$\sigma$ (ppm)	7.17	6.42	5.99 (454)	n/a	n/a
		MASE	0.11	0.09	0.08 (454)	n/a	n/a
Methanol-D4 (CD3OD)	2023 model	MAE (ppm)	9.46	5.29	3.08 (349)	n/a	n/a
		RMSE (ppm)	20.76	11.56	4.90 (349)	n/a	n/a
		MASE	0.22	0.12	0.07 (349)	n/a	n/a
		$\sigma$ (ppm)	19.71	11.47	4.90 (349)	n/a	n/a
	HOSE code	MAE (ppm)	4.12	3.66	3.15 (349)	n/a	n/a
		RMSE (ppm)	6.73	6.22	5.57 (349)	n/a	n/a
		$\sigma$ (ppm)	6.73	6.22	5.57 (349)	n/a	n/a
		MASE	0.10	0.09	0.07 (349)	n/a	n/a

n/a indicates that not enough data were available, numbers in brackets indicate the number of compounds used, deviating from the top header. The dataset does not contain molecules where HOSE model fails to predict shifts

**Fig. 6:**
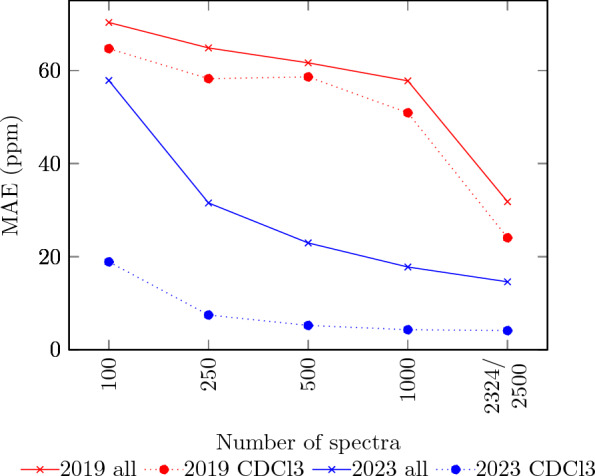
MAE of a $^{13}C$ NMR shift prediction, using increasing numbers of samples

Figure 6 shows the MAEs achieved by the two models trained with all data and CDCl3 only. It is clearly visible that the 2023 model outperforms the 2019 model. Furthermore, the CDCl3 predictions are not only better with each model than the predictions with all data, but the improvement with the 2023 model is higher than with the 2019 model.

To further verify our results, we have predicted the shifts of only CDCl3 spectra, but with all data used for training, using the 2019 model. For this, we have divided the CDCl3 spectra into four equal parts and trained models with all non-CDCl3 data and three of those parts. The fourth part was then used as a test set, predicting all shifts of it. The average errors of those for test sets were: MAE 2.04, RMSE 2.65, and $\delta$ 2.60. This confirms that the 2019 model is able to achieve good results also on the CDCl3 data alone, given enough data and that the good results of the 2023 model with CDCl3 data are not due to those data.

As before, we have also tested predictions leaving out molecules for which HOSE codes find no matches. The results for this are shown in Table 9. Similar to the non-solvent-specific prediction, there is no clear overall picture. For example, for DMSO-D6 the 2023 model now beats HOSE codes for 456 structures, whereas for CDCl3 this is not the case with 500 or 1000 structures.

**Fig. 7:**
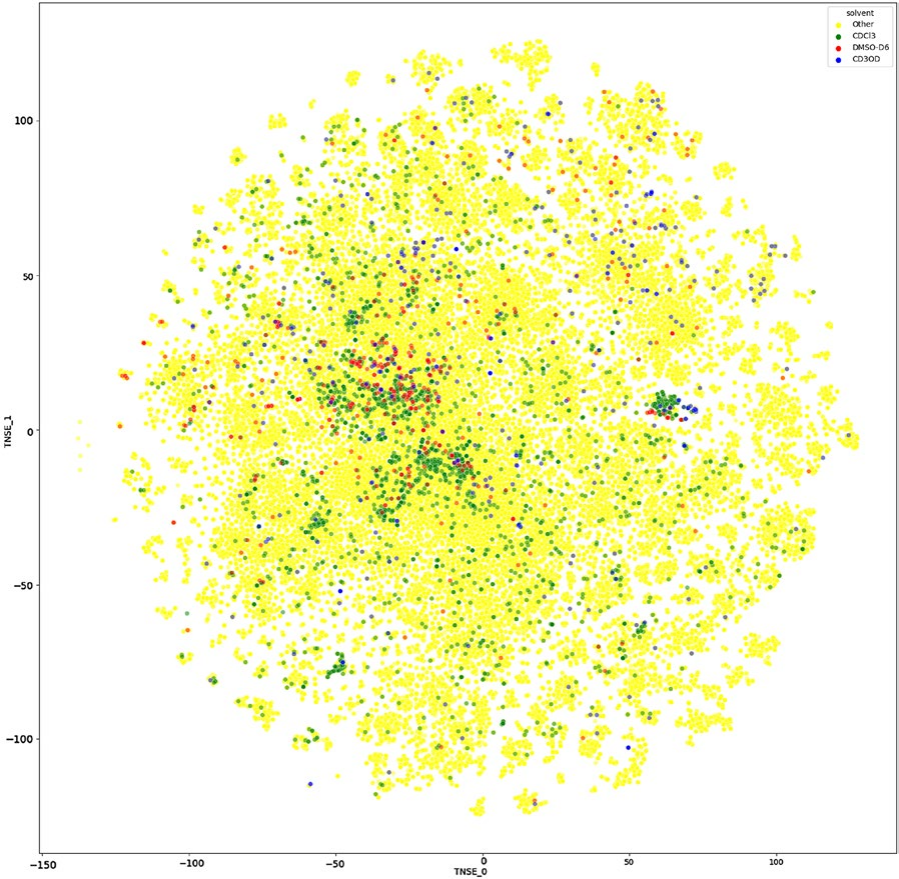
A plot of the compounds of nmrshiftdb2, distinguished by solvent, in chemical space. The calculation uses Extended Connectivity (ECFP) fingerprints to calculate descriptors and t-distributed stochastic neighbor embedding (t-SNE) for dimension reduction. The two major components are plotted. Using code from [25]

In order to verify that the distribution of the compounds is not dependent on solvents, we have plotted the compounds in a chemical space chart in Fig. 7. Here, all three solvents show a distribution similar to the overall database. It should be noted that all methods would be equally affected by any potential distortions.

## Discussion

In this paper, we have designed a new model based on message-passing graph neural networks for predicting chemical shifts. The new network is intended, in contrast to existing models, to work with small amounts of training data.

Testing this new model with $^{19}F$ data shows that it is possible to decrease the error rates significantly below the values of a standard deep learning model. Specifically, when trained on all $^{19}F$ data from nmrshiftdb2, our model achieves an MAE of 9.95 ppm, whereas the standard deep learning model only achieves an MAE of 57.82 ppm. In a similar fashion, it is also possible to improve the predictions on $^{13}C$ chemical shifts with a particular solvent. With the new model, we get an MAE of 4.5 ppm for all spectra, whereas the standard model only achieves 24 ppm. This clearly shows our model performs significantly better on smaller datasets.

Analysing Tables 4 and 6, we can conclude that sufficient results can be achieved by training the new model on roughly 1000 structures. This result is empirical and might depend on e.g. the diversity of the structures and the definition of a good prediction. For $^{13}C$, our model beats HOSE codes with 1000 structures, and for $^{19}F$, it is close to the HOSE code results with 957 structures. In that sense, the 1000 structures limit can be considered a rough threshold value where our model becomes useful. Of course, there could be models doing better with even smaller datasets.

Tests involving only molecules for which HOSE code predictions are possible (and which therefore could be considered more homogeneous datasets) improve results for the 2023 model with $^{19}F$ so that it is now better than HOSE codes with all structures. On the other hand, there is no clear picture for $^{13}C$ predictions. Overall, there are no conclusive results from those experiments.

Our model is only optimized for the prediction of $^{19}F$ chemical shift data and was used as-is for the other test cases. This means, that a more specialized model might perform better still for e.g. a particular nucleus or solvent. One way to improve performance would be to adjust the feature selection specifically for a dataset. The approach of using one model should work within solution NMR of organic compounds, where it could be useful to train a model specifically for a certain compound class. On the other hand, there are areas where this is unlikely to work, e.g. when predicting inorganic compounds or solids. The overall approach however could still prove useful as the availability of data is a problem often faced in research.

In this work, we have not tested all currently available models with different amounts of data. Some of them were published after the 2019 model and claim to have slightly better results using large datasets. Therefore, it is possible that they do better with small datasets, however, we expect the differences to the 2019 model to be negligible, as they have not been specifically built for and tested on smaller datasets.

## Conclusion

We have introduced a new machine learning model that can achieve more accurate NMR shift predictions than our previous model with a limited number of samples. When trained on $^{13}C$ NMR spectra, the model surpasses our previous model’s performance on datasets smaller than 2500 datapoints and outperforms HOSE codes when trained with datasets larger than 1000 datapoints. We also conducted tests on $^{19}F$ shifts and solvent-specific $^{13}C$ shifts. The new model consistently surpasses our previous model’s performance. Furthermore, with chloroform-specific $^{13}C$ shifts, the model achieves an MAE of less than 5 ppm. HOSE codes are still performing well in these cases, showing that there is potential for further enhancements by optimizing our new model for specific datasets. Our primary focus in this work was to highlight the performance improvement achieved by the generalized model. This approach could potentially be extended to other areas like inorganic compounds, although additional adjustments would likely be necessary to meet specific requirements. Regardless of our model’s performance, we have demonstrated the importance of assessing prediction methods using datasets of different sizes as a valuable quality measure.

## Scientifc contribution

We demonstrate the need to consider dataset size as a parameter in evaluating machine learning methods. We demonstrate this using NMR prediction as an example. We provide a new machine learning model improving prediction results for dataset sizes of 1000 to 2500 molecules.

### Supplementary Information

**Additional file 1: **The Python code for building and testing the 2023 model for standalone execution.

**Additional file 2: **The Python code for building and testing the 2023 model as a Jupyter notebook.

## Data Availability

The code of the project, together with explanations, is provided as a Jupyter notebook (Fluorine_GNN.ipynb). We also provide sd files with the ^13^C (nmrshiftdb2withsignals_13c.sd) respectively ^19^F data (nmrshiftdb2withsignals_19f.sd) used. Alternatively, the code and the data are available as a github repository at https://github.com/stefhk3/nmr-predict-small-quantity.
